# Update on the management of acute pancreatitis

**DOI:** 10.1097/MCC.0000000000001017

**Published:** 2023-01-23

**Authors:** Fons F. van den Berg, Marja A. Boermeester

**Affiliations:** a Amsterdam UMC location University of Amsterdam, Medical Microbiology & Infection prevention; b Amsterdam UMC location University of Amsterdam, Department of Surgery, Meibergdreef 9; c Amsterdam institute for Infection and Immunity; d Amsterdam Gastroenterology Endocrinology Metabolism, Amsterdam, The Netherlands

**Keywords:** necrotizing pancreatitis, post-ERCP pancreatitis, severe acute pancreatitis

## Abstract

This review provides insight into the recent advancements in the management of acute pancreatitis.

**Recent findings:**

Moderate fluid resuscitation and Ringer's lactate has advantages above aggressive fluid resuscitation and normal saline, respectively. A normal “on-demand” diet has a positive effect on recovery from acute pancreatitis and length of hospital stay. A multimodal pain management approach including epidural analgesia might reduce unwarranted effects of opiate use. A more targeted use of antibiotics is starting to emerge. Markers such as procalcitonin may be used to limit unwarranted antibiotic use. Conversely, many patients with infected necrotizing pancreatitis can be treated with only antibiotics, although the optimal choice and duration is unclear. Delay of drainage as much as is possible is advised since it is associated with less procedures. If drainage is required, clinicians have an expanding arsenal of interventional options to their disposal such as the lumen-apposing metal stent for transgastric drainage and (repeated) necrosectomy. Immunomodulation using removal of systemic cytokines or anti-inflammatory drugs is an attractive idea, but up to now the results of clinical trials are disappointing. No additional preventive measures beside non-steroidal anti-inflammatory drugs (NSAIDs) can be recommended for post-endoscopic retrograde cholangiopancreatography (ERCP) pancreatitis.

**Summary:**

More treatment modalities that are less invasive became available and a trend towards less aggressive treatments (fluids, starvation, interventions, opiates) of acute pancreatitis is again emerging. Despite recent advancements, the pathophysiology of specific subgroup phenotypes is still poorly understood which reflects the disappointing results of pharmacological and immunomodulatory trials.

## INTRODUCTION

Acute pancreatitis (AP) is a (initially) sterile inflammation of the pancreas that evokes a systemic inflammatory response syndrome (SIRS) with large heterogeneity in terms of severity. Around 80% of patients experience mild symptoms that merely require supportive therapy with fluids, analgesia, and diet resumption. Nevertheless, a small fraction of patients is being admitted to intensive care units (ICUs) within the first days due to an overwhelming SIRS response causing persistent (multiple) organ failure. Besides supportive care until the inflammation resides, no specific therapies are yet available to mitigate or prevent this.

Despite recent treatment approaches such as the surgical and endoscopic step-up approaches [1,2] that have been adopted in most practices, infected (peri-) pancreatic necrosis remains a challenge for clinicians. A recent nationwide analysis shows that mortality in patients with acute pancreatitis who had been admitted to Dutch ICUs remained unchanged for the last two decades (average 23% hospital mortality) [3^▪^]. However, all-cause 1-year mortality was higher before 2010 (up to the publication date of the PANTER trial [2]) for patients with late mortality (after 14 days), suggesting that mainly treatment of late complications has improved.

This review presents an overview of recent clinical trials, while also focusing on novel insights, that will most likely affect the management of acute pancreatitis (Table 1). 

**Box 1:**
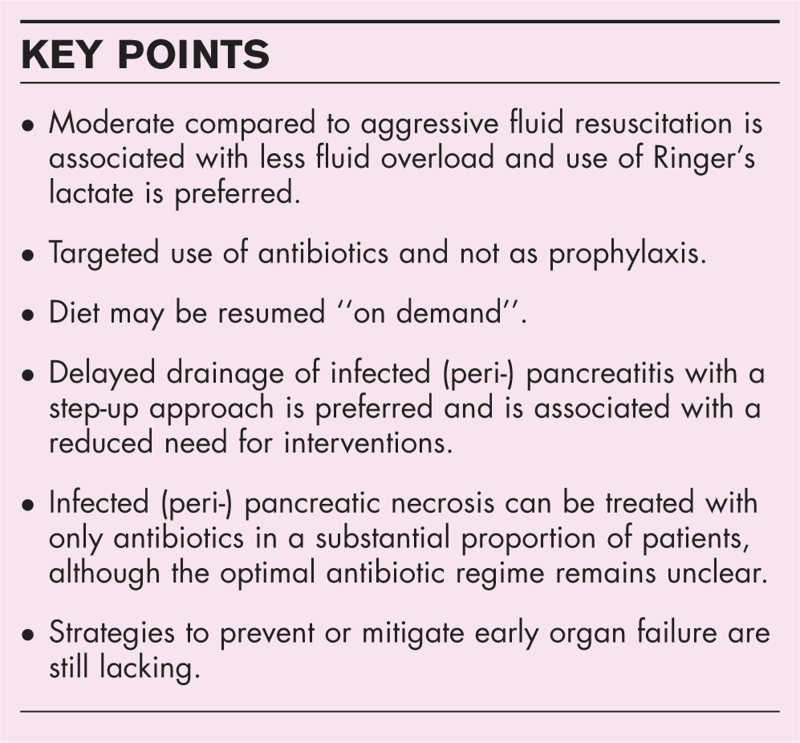
no caption available

**Table 1:** Recommendations based on recent literature

Early management	
Fluids	Administer a moderate fluid resuscitation (10 ml/kg bolus in case of hypovolemia, followed by 1.5 ml/kg/h) using Ringers lactate
Nutrition	Resume an on-demand diet as soon as tolerated. Consider feeding tube placement if diet has not been resumed after three days
Pain management	Multimodal approach that combines paracetamol, metamizole and simple opiates. When insufficient consider epidural analgesia
Prophylaxis	Prophylactic antibiotics, pre, pro- and postbiotics can currently not being recommended

Management of complications	
Antibiotics	Carbapemens remains the first choice of empiral antibiotics for treatment of infected necrosis, although robust evidence is lacking Procalcitonin may be used as guidance when antibiotic therapy is considered to reduce unnecessary antibiotic use
Infected necrosis	Drainage of infected necrotic collections should be delayed and treated with antibiotics alone if possible Modality of drainage (percutaneous, transgastric plastic of lumen-apposing metal stents) should be discussed interdisciplinary and depends on the location and extent of the collections, and local availability and expertise
Intra-abdominal hypertension	Neostigmine treatment should be considered for patients admitted to the ICU with intra-abdominal hypertension
Biliary necrotizing pancreatitis	Patients with biliary necrotizing pancreatitis should be closely monitored following discharge and offered cholecystectomy when collections have been resolved
Persistent organ failure	Extracorporeal cytokine removal might be considered as last resort for persistent organ failure in selected cases

## EARLY MANAGEMENT

### Fluids

Goal-directed fluid resuscitation is the cornerstone of initial management of acute pancreatitis, although based on very limited evidence. A Spanish trial randomizing patients with acute pancreatitis between aggressive goal directed and moderate resuscitation was halted because patients in the aggressive group experienced more fluid overload (20.5% vs. 6.3%), while not preventing progression to moderate or severe acute pancreatitis [4^▪▪^]. Another trial comparing aggressive vs. normal resuscitation did not show a difference in clinical outcomes [5].

Although evidence is scarce, comprising small RCTs with irrelevant clinical primary outcomes, the international guidelines advocate the use of Ringers lactate. The single-center trial of Lee *et al.*[6^▪^] meant to put an end to the debate on choice of fluids. They have randomized 121 patients between goal-directed fluid resuscitation with normal saline (NS) or Ringer's lactate (RL). SIRS score at 24 h after administration as primary outcome of the study is not different between NS (23.3%) and RL (27.9%). Secondary outcomes differ significantly in favor of RL, such as median length of hospital stay (3.5 [2–5.9] vs. 4.6 [3–7.4] days), ICU admission (9.8% vs. 25%), and pancreatic necrosis (6.6% vs. 15%). The authors have excluded two thirds of patients with acute pancreatitis. The most common reasons for exclusion were logistical reasons (40%) and liver, renal or cardiac insufficiency (35.2%). Of the patients with mild acute pancreatitis, around 20% progressed to moderate or severe acute pancreatitis, which is comparable to the literature. A recent systematic review confirms that Ringers reduces ICU admission and hospital stay [7]. Recent randomized unblinded trials of lower quality show reduced CRP levels and less organ failure with RL [8,9]. Despite these major limitations, these studies strengthen the current recommendations regarding the use of RL. Future studies should limit their exclusion criteria and use a clinically relevant (composite) endpoint, for example major complications such as new onset organ failure and pancreatic necrosis. Goal-directed aggressive fluid therapy will most likely be abandoned and be replaced by moderate resuscitation (10 ml/kg bolus in case of hypovolemia, followed by 1.5 ml/kg/h). In patients with severe acute pancreatitis, novel fluid administration strategies such as colonic fluid resuscitation [10] and pulmonary artery catheter- guided resuscitation [11] show favorable results but their value remains unclear.

### Nutrition

Nutrition remains a highly debatable subject in early pancreatitis management. Starvation in acute pancreatitis is driving pathogenic processes such as gut failure and bacterial translocation. A recent Spanish trial has compared conventional fasting until biochemical and clinical improvement to immediate resumption of a solid diet at admission [12^▪^]. This trial has randomized 131 patients with mild/moderate acute pancreatitis; 99% of patients in the early feeding group resumed their diet immediately while this took a mean of 2.8 (1.7) days in the conventional feeding group. Only 1 patient (1.4%) in the early feeding group experienced intolerance for diet vs. 13 (21.6%) in the conventional group. The primary outcome length of hospital stay was significantly lower in the early feeding group [mean 3.4 (1.7) vs. 8.8 (7.9) days]. Also, progression to moderate AP, complications, and IC admissions are lower in the early feeding group. Rai *et al.*[13] have conducted a similar randomized controlled trial (RCT) with patients with moderate or severe acute pancreatitis. Mean length of hospital stay was 6.3 (3.5) vs. 7.3 (3.4) days in favor of on-demand oral feeding. The prolonged hospital stay in a conventional feeding strategy is not surprising since length of stay is driven by a fasting period and build-up of the diet. Therefore, length of hospital stay may not be clinically relevant here. Also, both studies use food intolerance as secondary outcome, which may introduce interventional bias since patients may assume that starvation is a necessary part of their treatment and therefore later resume their diet. Finally, both studies do not mention the actual caloric intake per day, which is a better reflection of the nutritional status. Two recent meta-analyses confirmed that early diet resumption is associated with short hospital stay [14,15].

In our clinical practice, generally “the nil per os” strategy has been replaced for some years now by an on-demand oral diet that is offered on admission. In our opinion, nasogastric tube feeding (or nasojejunal tube in case of gastropareses) should be considered if an oral diet has not been resumed within three days. Parental feeding should be reserved for patients that are persistently intolerant to enteral feeding (i.e., due to paralytic ileus). Future research should focus on specialized (or better personalized) nutrition tailored to the disease severity and phase and to the patient's needs.

### Pain management

Pain management in pancreatitis is poorly studied and a substantial variation exists between and within clinical practices. Traditionally, pain management is cornered on the use of opiates although nowadays a multimodal approach paracetamol, non-steroidal anti-inflammatory drugs (NSAIDs), metamizole, opiates, ketamine and epidural analgesia is commonly used. Two recent meta-analyses have appeared, identifying 6 and 12 RCTs, respectively [16,17]. Both meta-analyses indicate that NSAIDs and opiates are equally effective, but a substantial paucity of data exists. Epidural analgesia may also be an effective opiate sparing modality, although only one RCT has been identified. A recent retrospective analysis of 352 patients with severe acute pancreatitis admitted to the ICU of a Chinese hospital suggests thoracic epidural analgesia may provide protection against adult respiratory distress syndrome (ARDS), acute kidney injury (AKI), and even mortality [18^▪^]. An unblinded trial has randomized patients between hydromorphone patient-controlled analgesia (PCA) and intramuscular pethidine and showed no difference in pain relief, but an overall worse outcome in the hydromorphone-PCA group resulting in premature termination of the trial [19].

Although based on low quality and low sample size evidence, a multimodal approach that combines paracetamol, metamizole and simple opiates is recommended. When insufficient, an Acute Pain Service, nowadays available in most hospitals, can be consulted for additional modalities. Epidural analgesia is still infrequently used but shows promise as an opiate-sparing alternative, depending on in-house availability.

### Prophylactic antibiotics

Although there are many, often contradiction, studies, current guidelines advise against the routine use of prophylactic antibiotics for treatment of acute pancreatitis since there is no clear benefit. A recent meta-analysis of seven studies again confirmed this by showing that prophylactic carbapenems, the most widespread used treatment for infected necrosis, reduces urinary tract infections, pneumonia and bacteremia, but did not show a beneficial effect on infected necrosis, mortality and other clinically important outcomes [20]. Also, that meta-analysis has included two retrospective studies that may have biased the results. Although continuously under debate, it seems that prophylactic antibiotics may do more harm than good and therefore is still not recommended.

### Pre, pro- and postbiotics

For the last decades it becomes more apparent that the gut microbiome is an important disease modifier in severe inflammatory conditions such as acute pancreatitis. Nevertheless, trials that were aimed to reduce the gut pathobiome (collection of pathogens) though antibiotics or enhance the commensal microbiota with pre or probiotics did not lead to an effective and safe prophylactic treatment for administration in the early, hyperinflammatory phase of acute pancreatitis. Two recent randomized trials showed that synbiotics (Bifilac) in moderate and severe pancreatitis and probiotics (Bacillus subtilis and Enterococcus faecium) in mild pancreatitis, respectively, reduced the length of hospital stay but did not affect clinically important outcomes [21,22]. Chen *et al.*[23] have randomized 49 patients with severe acute pancreatitis between soluble dietary fibers (polydextrose) or control. Feeding intolerance, defined as the need to stop or reduce enteral nutrition, is reduced from 72.73% to 29.17% with soluble dietary fibers. However, there was no blinded outcome assessment which introduces important risk of bias.

Postbiotics, bacterial products that are produced from fibers, are attractive targets because of their endogenous nature and safety profile. A recent preclinical study showed promise for the use of butyrate, a short chain fatty acid, in the prevention of severe complications in mice [24^▪^]. A proof-of-concept trial using micro-encapsulated tributyrin (a butyrate prodrug) as prophylaxis in patients with acute pancreatitis is currently being designed.

### Prevention of post-ERCP pancreatitis

A lot of effort has been invested to reduce the incidence of post-endoscopic retrograde cholangiopancreatography (ERCP) pancreatitis (PEP). As mentioned, it is now standard of care to administer rectal NSAIDs. Moreover, a pancreatic duct (PD) stent is placed in case of unintentional PD cannulation. A Japanese propensity-score matched analysis shows that low dose diclofenac (25 mg) in patients with a body weight <50 kg is not effective [25]. A large international prospective observational study has shown no protective effect of chronic statin and aspirin use [26]. Besides the routine administration of NSAIDs, prophylactic hyperhydration has gained a lot of attention recently. A large multicenter RCT has randomized patients with moderate or high risk for PEP between standard of care and an aggressive hydration protocol using Ringer's lactate. Although mean fluid administration in the first 24 h is significantly higher in the aggressive hydration compared to control (3562 ml vs. 400 ml), comparable PEP incidence is found (8% vs. 9%) [27^▪^]. A Japanese trial has found comparable PEP incidence for rectal diclofenac, PD stenting, and a combination of both [28]. However, at a very low incidence of PEP (1.6%), the study was likely underpowered to detect a potential difference.

No additional prophylactic measures other than a high dose of rectal NSAIDs (100 mg diclofenac) and pancreatic duct stenting in case of unintentional PD cannulation can currently be recommended.

## MANAGEMENT OF COMPLICATIONS

### Antibiotic treatment

The prescription of antibiotics is common during acute pancreatitis; up to two third of patients are administered antibiotics during the disease course, often without a culture- or radiologically proven infection. Clinicians who are confronted with fever and elevated inflammatory parameters early in the disease course initiate empirical antibiotics due to the inability to discriminate between SIRS and infection.

Procalcitonin (PCT) is a useful biomarker that is elevated in bacterial infection but not in inflammation. A UK single-center RCT has randomized 260 patients between PCT-guided antibiotic treatment or usual care [29^▪▪^]. In the intervention group, PCT was measured at days 0, 4 and 7, and thereafter weekly or when a clinical decision was going to be made to initiate or stop antibiotics. When a PCT test indicated >1 ng/ml, the advice was to initiate antibiotics; at <1 ng/ml, the advice was to stop or not to initiate antibiotics. Clinicians deviated from the algorithm in 24 instances, mostly initiating or continuing antibiotics despite a negative PCT test (79%). In the PCT-guided intervention group, therapeutic antibiotic prescription was significantly lower compared to the usual care group (41% vs. 60%). The infection and adverse advents rates were comparable among groups. PCT-guided care may reduce unwarranted antibiotic use without risking severe complications. However, in the subgroup of patients with moderate or severe acute pancreatitis there was no difference in antibiotic prescription among groups, suggesting that PCT-guide care is mainly effective in reducing antibiotic use in the early hyperinflammatory phase. The results do not give insight in what type of patients and in what phase of the disease clinicians decide to adhere or deviate from the algorithm. The indication, choice and duration of antibiotics administration is not described by the authors. A recent Spanish prospective study showed that PCT-guided antibiotic therapy on admission was associated with infectious complications [30]. It does not seem logical to use PCT levels on admission to guide antibiotic treatment for infections that usually occur after weeks.

Based on these studies, PCT testing will most likely be implemented in pancreatitis care globally, stimulated by recent Antibiotic Stewardship Programs. Future efforts to limit unwarranted antibiotic use must address the value of algorithms in specific disease phenotypes and disease phases. Moreover, the effects of such algorithms have yet to be evaluated in countries that are restrictive in antibiotic use such as the Netherlands and Scandinavian countries, as in such clinical practices this may even result in an increase instead of decrease in antibiotic administration. Carbapenems such as meropenem and imipenem are most frequently used as empirical antibiotics because of limited evidence showing penetration of pancreatic tissue, however practice variation is large and clear guidelines are lacking.

### Management of severe local complications

In the last decades, significant progress has been made in the treatment of infected necrotizing pancreatitis. A range of new interventional procedures have become available, such as the video-assisted retroperitoneal debridement (VARD), transgastric drainage, and necrosectomy. The step-up approach (surgically [2] or endoscopically [1]) has become the global standard of care. The 5-year follow up of a trial comparing the endoscopic and surgical step-up shows comparable outcomes, although less pancreatic fistulas with the endoscopic approach [31^▪^].

It has been recommended to wait for the development of walled-of-necrosis before drainage to prevent complications. Nevertheless, early drainage can potentially prevent a gradually increasing inflammatory response or cytokine storm. In the Dutch POINTER trial, patients with infected necrotizing pancreatitis have been randomized between early (mean 24 days) or late drainage (mean 34 days) [32^▪▪^]. The Comprehensive Complication Index did not differ among groups but the postponed group overall received less interventions (mean 2.6 vs. 4.4). More importantly, 39% of the postponed group did not receive any intervention at all and were treated with antibiotics alone, while all patients in the early drainage group received an intervention.

The AXIOMA study has added a third treatment arm including patients being drained using a lumen-apposing metal stent [33^▪^]. The need for transluminal necrosectomy following drainage was equal compared to patients drained with plastic stents in the POINTER trial. The same study group has retrospectively reviewed patients undergoing endoscopic drainage of symptomatic sterile necrotic collections and have found a high success rate (87%) but also a high rate of iatrogenic secondary infections (73%) [34].

A further course towards a more conservative approach to infected necrotizing pancreatitis using only antibiotics is justified by these recent studies. The optimal choice and duration of antibiotics and radiological follow-up of conservative treatment is unclear and is in need of more data and the development of clear guidelines. The choice of interventional modality is mainly dictated by the location of fluid/necrosis collections and in-house availability of expertise. Optimal treatment warrants centralization of care and multidisciplinary consultation. A sterile necrotic collection only requires drainage when symptomatic and suspicious of undiagnosed infection of that collection, counterbalanced by a high iatrogenic infection rate.

Neostigmine treatment reduces the intra-abdominal pressure and increases stool volume in patients admitted to the ICU. Intra-abdominal hypertension (IAH) is believed to be associated with worse outcome, although no effect on clinically important outcome measures such as new-onset organ failure, mortality, or the need for surgical decompression has been shown. Therefore, the direct benefit for patients of interventions to lower IAH remains unclear [35].

The standard for mild biliary pancreatitis is a same-admission cholecystectomy, but there is no consensus regarding the optimal timing of cholecystectomy for patients with necrotizing pancreatitis. A large retrospective analysis shows that cholecystectomy was performed at a median of over 3 months following discharge [36^▪^]. Early (<8 weeks) cholecystectomy might reduce biliary recurrences, however due to the retrospective nature there remains a strong need for a (randomized) trial.

### Immunomodulation

Severe acute pancreatitis is characterized by hyperinflammation in the early phase that leads to (multiple) organ failure and high mortality rates. Modulation of this hyperinflammatory response by removing components of the cytokine storm is a (theoretically) attractive approach that is getting more attention lately. Two recent studies have investigated filtration of blood to remove cytokines [37,38]. A meta-analysis of 17 studies shows that high-volume hemofiltration reduces short term (<4 weeks) but not long-term mortality [37]. A small observational study reports 16 patients with severe acute pancreatitis being treated with Cytosorb, an extracorporeal blood purification device that selectively absorbs cytokines [38]. In this small series, the treatment improves hemodynamics in comparison with 32 APACHE-II-score matched patients. Therapeutic plasma exchange is sometimes used as a last resort to treat patients with refractory multiple organ failure. Case series data show that despite a temporary improvement of hemodynamics, almost all patients still die within 28 days [39]. Future innovations may enable us to better treat or even prevent organ failure.

Infected necrotizing pancreatitis most often occurs late in the disease course. Immunoparalysis due to prolonged preceding hyperinflammation may significantly contribute to this late infection. Following a successful pilot study, a Chinese group has performed a large randomized placebo-controlled trial using Thymosin alpha 1, an immunomodulatory natural occurring peptide in the human thymus which is currently utilized in immunocompromised patients and as enhancer of vaccine response [40]. In contrast to the pilot study results, the RCT shows no difference in clinical outcomes between the intervention and placebo group.

Prophylactic administration of NSAIDs to prevent post-ERCP pancreatitis has become routine care. The presumed working mechanism, reduction of the proinflammatory response through cyclooxygenase (COX) inhibition, may also be a viable option in patients with acute pancreatitis who have a high risk of developing organ failure. A large US retrospective study based on ICD-9 codes suggests that any prior NSAID use is inversely associated with organ failure [41]. A US RCT has randomized 42 patients with SIRS but without organ failure between a 100 mg loading dose of rectal indomethacin, followed by five doses of 50 mg every 8 h, or placebo [42]. SIRS score as primary outcome and other markers clinical outcomes are comparable among groups; no beneficial effect is seen.

These disappointing results may very well reflect our fundamental lack of understanding of the pathophysiological mechanisms that underlie (early) organ failure. Recently, using multiomics techniques, 4 subtypes of molecular endotypes have been identified in patients with acute pancreatitis, resembling generalizable endotypes seen in ARDS patients [43^▪^]. Notably, all patients with persistent organ failure clustered in subtype A despite adding clinical data to the model. Although exploratory, more such studies are needed to study specific disease processes that may be manipulated in future clinical trials.

## CONCLUSION

Early management of acute pancreatitis is continuously improving and includes fluid resuscitation, nutrition, and analgesia. Recent trends are towards a more conservative approach using less fluids, less opiates, and delay interventions as much as possible. Trials that are focused on reducing (early) organ failure through immunomodulation still produce disappointing results. A more fundamental understanding of the early inflammatory response is therefore needed.

## Acknowledgements

*None.*

### Financial support and sponsorship

*None.*

### Conflicts of interest

*There are no conflicts of interest.*

## REFERENCES AND RECOMMENDED READING

Papers of particular interest, published within the annual period of review, have been highlighted as:

▪ of special interest
▪▪ of outstanding interest
